# Novel variants of *NEK9* associated with neonatal arthrogryposis: Two case reports and a literature review

**DOI:** 10.3389/fgene.2022.989215

**Published:** 2023-01-04

**Authors:** Fang Liu, Liying Dai, Zhi Li, Xiaowei Yin’s

**Affiliations:** ^1^ Department of Pediatrics, NICU, the 980th Hospital of the People’s Liberation Army Joint Service Support Force, Bethune International Peace Hospital, Shijiazhuang, China; ^2^ Department of Neonatology, Anhui Children’s Hospital, Hefei, China

**Keywords:** NIMA-related kinase 9 (NEK9), arthrogryposis, recessive, mutation, clinical

## Abstract

**Objective:** Pathogenic variants in *NEK9* (MIM: 609798) have been identified in patients with lethal congenital contracture syndrome 10 (OMIM: 617022) and arthrogryposis, Perthes disease, and upward gaze palsy (APUG and OMIM: 614262). The shared core phenotype is multiple joint contractures or arthrogryposis. In the present study, three novel variants of *NEK9* associated with neonatal arthrogryposis were reported.

**Methods:** The clinical data of two premature infants and their parents were collected. The genomic DNA was extracted from their peripheral blood samples and subjected to trio-whole-exome sequencing (trio-WES) and copy number variation analysis.

**Results:** Using trio-WES, a total of three novel pathogenic variants of *NEK9* were detected in the two families. Patient 1 carried compound heterozygous variations of c.717C > A (p. C239*741) and c.2824delA (p.M942Cfs*21), which were inherited from his father and mother, respectively. Patient 2 also carried compound heterozygous variations of c.61G > T (p. E21*959) and c. 2824delA (p. M942Cfs*21), which were inherited from his father and mother, respectively. These variants have not been previously reported in the ClinVar, HGMD, or gnomAD databases.

**Conclusion:** This is the first report about *NEK9-*related arthrogryposis in neonatal patients. The findings from this study suggest that different types of mutations in *NEK9* lead to different phenotypes. Our study expanded the clinical phenotype spectrum and gene spectrum of *NEK9*-associated arthrogryposis.

## 1 Introduction

Arthrogryposis is frequently used to describe multiple congenital contractures that impact more than two areas of the body. Arthrogryposis is not a specific diagnosis but instead a clinical finding, and it is a feature of more than 300 different disorders. The prevalence of arthrogryposis is 1 in 3,000 live births (Bamshad et al., 2009; Kowalczyk et al., 2016; Fahy and Hall, 1990). Arthrogryposis is further a group of clinical mitotic control from scattered single gene mutations, chromosomal disorders, and mitochondrial disorders (Bamshad et al., 2009).

*NEK9* encodes a member of the NIMA family of serine/threonine protein kinases, which is triggered in mitosis and, in turn, triggers other family members during mitosis (O'Connell et al., 2003). The functions of *NEK9* in the spindle assembly and centrosome separation (Kaneta and Ullrich, 2013) are essential in the regulation of mitosis. Dysfunction of *NEK9* leads to multiple system impairments, including ocular, skeletal, and central nervous system anomalies and various tumors (Sheppard et al., 2020; Panchal and Evan Prince, 2022). Pathogenic variants in *NEK9* were identified in the fetus with lethal congenital contracture syndrome 10 (OMIM: 617022) and in children with arthrogryposis, Perthes disease, and upward gaze palsy (APUG and OMIM: 614262). To our knowledge, only eight cases have been fully reported (Alkuraya, 2010; Shaheen et al., 2015; Casey Jillian et al., 2016; Stals Karen et al., 2018; Deden et al., 2020).

In this study, we reported two unrelated neonates from non-consanguineous families with healthy parents. The patient presented with arthrogryposis (HP: 0012453), pyloric stenosis (HP: 0002021), atrial septal defect (HP: 0001631), and pulmonary stenosis (HP: 0001642). Using trio-WES, we identified two novel compound heterozygotic variants of *NEK9* (c.717 C > A, p. C239*741 and c.2824delA, p.M942Cfs*21; c.61 G > T, p. E21*959 and c.2824delA, p.M942Cfs*21). Our findings expand the clinical phenotype spectrum and the gene spectrum of *NEK9*-associated arthrogryposis.

## 2 Subjects and methods

### 2.1 Subjects

#### 2.1.1 Patient 1

Patient 1 was a boy, who was delivered by cesarean section with maternal placental abruption. Gestational age was only 32^+2^ weeks, birth weight: 1680 g (< 3rd), length: 40 cm (< 3rd), and head circumference: 30 cm (< 3rd). His mother was 31 years old, and the pregnancy was uneventful. His father was 35 years old. The parents were unrelated and in good health; there was no family history of congenital malformations or hypertension. After birth, the premature baby was hospitalized in the NICU of the local maternal and child health hospital. He was transferred to our hospital on the second day after birth due to respiratory distress and pulmonary hypertension. He was treated with exogenous pulmonary surfactant, blood plasma, and mechanical ventilation for 7 days and then was extubated to CPAP for another 14 days. Five weeks later, his saturation could reach more than 90% in room air. Cranial ultrasound was performed routinely on days 3 and 7 after admission, and he was detected as having severe intraventricular hemorrhage (grade Ⅲ) with ventricular dilatation (10 mm–15 mm). The hemorrhage was absorbed after three weeks with the bilateral ventricle mild dilatation. Other clinical manifestations included normal scrotal pigmentation, normal penis size, and bilateral testicular descent. Echocardiography revealed patent foramen ovale, mild pulmonary stenosis, and mild-to-moderate pulmonary hypertension. Liver, kidney, and thyroid functions were normal. Serum lipid and myocardial enzyme levels were in the normal range. Routine blood, urine, and stool test results were normal. Normal plasma amino acid and urinary orotic acid levels were demonstrated.

**FIGURE 1:**
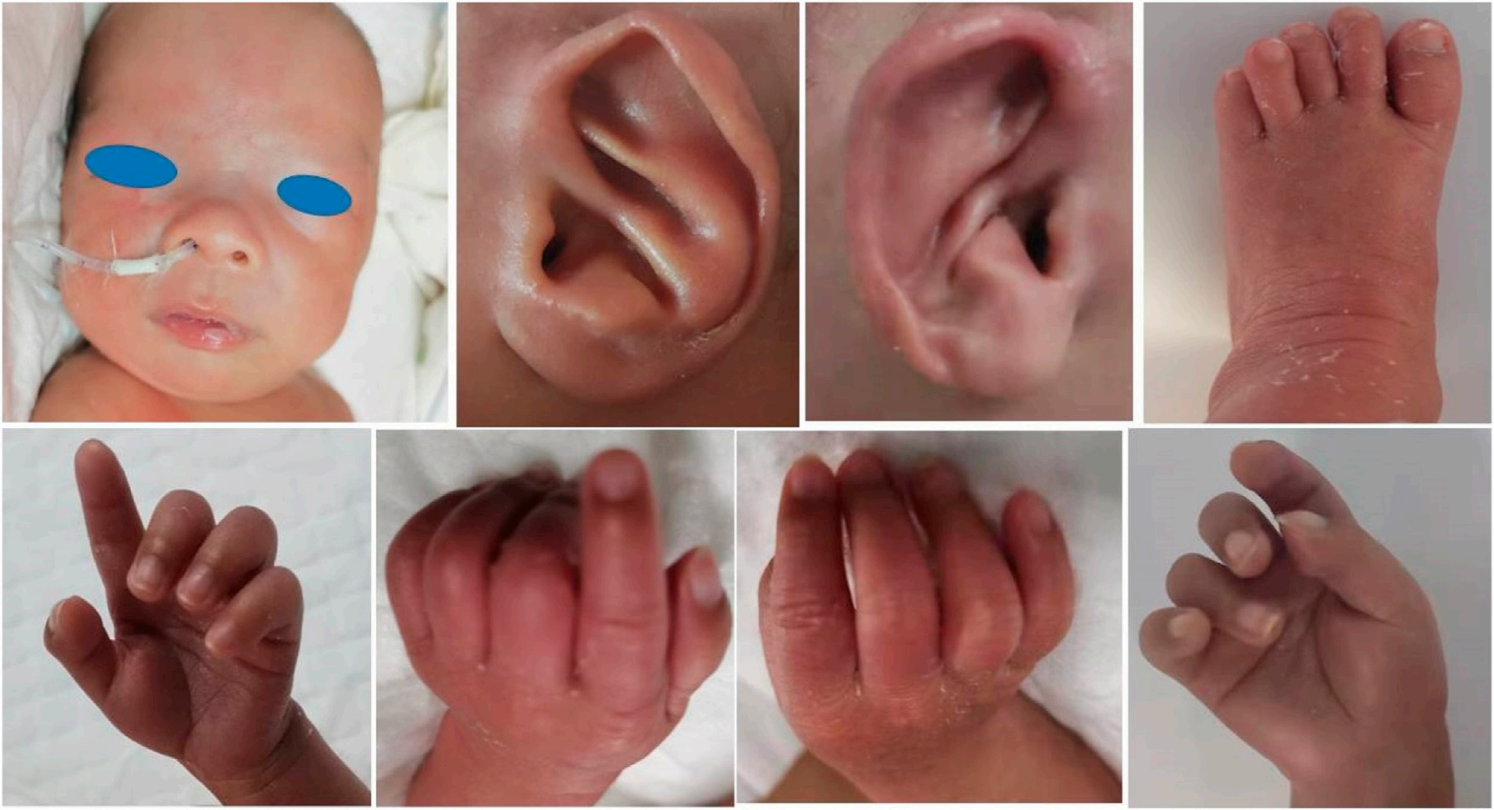
Patient Ⅰ: sparse eyebrows and hair, prominent forehead, wide eye distance, wide nose bridge, and prominent antihelix stem. His bilateral third, fourth, and fifth fingers were camptodactyly. Mild deformity of the left toe was also present.

Special appearance: from the beginning of admission, doctors noticed that the boy’s neck was stiff; the pediatrician and the physical therapist also confirmed the presence of a moderate form of arthrogryposis involving the elbows and knees, and his bilateral third, fourth, and fifth fingers were camptodactyly (Figure 1). After tube feeding, he presented with abdominal distension, vomiting, and larger gastric retention. Abdominal X-ray showed a huge stomach bubble, and pyloric stenosis was suspected in gastrointestinal angiography and abdominal ultrasonography. On the 32nd day after birth, the patient underwent pyloric hypertrophy resection surgery. He was given tube feeding 4 days after the operation, until complete enteral feeding.

#### 2.1.2 Patient 2

Patient 2 was a boy, delivered by cesarean section with maternal polyhydramnios. Gestational age was only 34^+2^ weeks, birth weight: 2,700 g (50th–90th), length: 43 cm (10th), and head circumference: 33 cm (50th–90th). The parents were unrelated and in good health; there was no family history of congenital malformations or hypertension. After birth, the premature baby was hospitalized in the NICU of the local hospital and was diagnosed with sepsis at 6 days of age, and he received regular antibiotic treatment. Echocardiography showed atrial septal defect and pulmonary artery stenosis. Brain magnetic resonance imaging showed that the T2WT signal in the white matter of the bilateral cerebral hemispheres was high, and the EEG was normal. After birth, the pediatrician noticed that the child’s muscle tone was significantly increased, his neck was stiff, and the passive extension of his elbows, hips, and knees was limited. The patient was discharged after 2 months of birth, and he was still unable to suck and required tube feeding.

## 3 Method

### 3.1 Sample collection

The study was approved by the Ethics Committee of Bethune International Peace Hospital and Anhui Provincial Children’s Hospital. The parents of the boys gave written informed consent for clinical examination, genetic analysis, and data publication. A peripheral blood sample was collected from the two boys and their parents. Genomic DNA of the two boys and their parents were obtained from the peripheral blood leukocytes using the Blood Genome Column Medium Extraction Kit (Kangweishiji, China) for trio-whole-exome sequencing (trio-WES) and copy number variation (CNV) analysis.

### 3.2 Genetic analysis

Genomic DNA of the two boys and their parents were obtained from the peripheral blood leukocytes using the Blood Genome Column Medium Extraction Kit (Kangweishiji, China) for trio-whole-exome sequencing (trio-WES) and copy number variation (CNV) analysis. DNA pieces were hybridized and exome enrichment was captured using xGen Exome Research Panel v1.0 (IDT, Iowa, United States) which contains 429,826 probes, targets 39 Mb protein-coding region, and overlaps 51 Mb of end-to-end tiled probe space. Illumina NovaSeq 6000 series sequencer (PE150) was operated, and more than 99% of the target sequence was sequenced. Sequencing raw data were processed by FASTQ for removing adapters and filtering low-quality reads. The paired-end reads were aligned to the Ensemble GRCh37/hg19 reference genome using Burrows–Wheeler aligner (BWA). Base quality score recorrecting and variant calling were conducted using GATK. SNPs and indels were screened in accordance with the sequencing depth and variant quality. Variant annotation and pathogenicity prediction were processed based on the databases for minor allele frequencies (1000 Genomes, dbSNP, ESP, ExAC, and gnomAD), and the pathogenicity of genetic variants was predicted by bioinformatics tools such as PolyPhen, MutationTaster, REVEL, and CADD. Finally, variants were classified according to the American College of Medical Genetics and Genomics (ACMG) guideline (Richardset al., 2015).

Detection of CNV: raw image files were processed by applying Bcl To FASTQ (Illumina) for raw data generation. The reads were then mapped to the GRCh37/hg19 human reference genome using BWA software. Variant calling for CNVs of 100 KB and above in length was detected using Chigene independently developed software packages for CNV detection, and the candidate CNVs were filtered and detected using public CNV databases (Decipher, ClinVar, OMIM, DGV, and ClinGen). The biological harm and related phenotypes of CNVs were assessed by annotated information and frequency databases depending on ACMG practice guidelines and CNV diagnostic guidelines (Richards et al., 2015; Riggs et al., 2022).

## 4 Results

### 4.1 Results of genetic analysis

By trio-WES, a total of three novel pathogenic variants of *NEK9* were detected from the two families. Patient 1 carried compound heterozygous variations: c.717 C > A (p. C239*741) and c.2824delA (p. M942Cfs*21), which were inherited from his father and mother, respectively. These variants have not been reported in the ClinVar, HGMD, and public databases (gnomAD v2.1.1, http://gnomad.broadinstitute.org/ and the 1000 Genomes Project, http://www.internationalgenome.org). According to ACMG guidelines, we confirmed the two variants to be likely pathogenic (PVS1+PM2 and PVS1-Strong + PM2+PM3) (Richards et al., 2015; Harrison et al., 2019). Patient 2 also carried compound heterozygous variations, which were c.61 G > T (p. E21*959) and c.2824delA (p.M942Cfs*21) that were inherited from his father and mother, respectively. This variant of c.61 G > T has also not been reported in the ClinVar, HGMD, and public databases (gnomAD v2.1.1, http://gnomad.broadinstitute.org/ and the 1000 Genomes Project, http://www.internationalgenome.org). According to ACMG guidelines, we confirmed the variant to be pathogenic (PS2+PM1+PM2+PP2+PP3) (Richards et al., 2015; Harrison et al., 2019).

Moreover, we did not detect pathogenic or likely pathogenic variants in genes known to be associated with the clinical features of probands using trio-WES.

**FIGURE 2:**
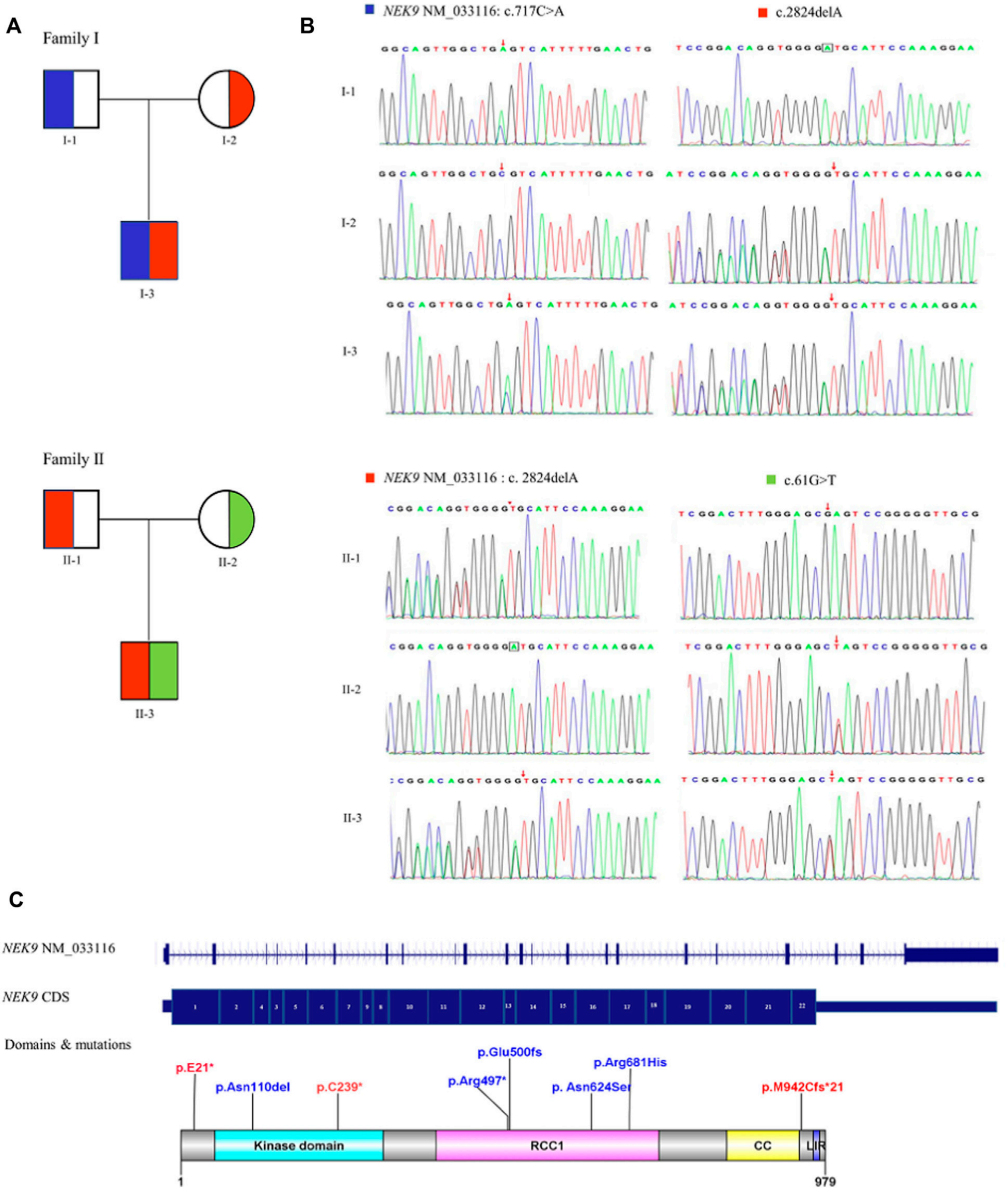
Diagram of *NEK9* mutations and gene structure. **(A)** Pedigree diagrams of two families with *NEK9* mutations. Each color corresponds to a novel variant of *NEK9*. **(B)** Sanger sequencing results of the diagnostic variants in the two families. **(C)** Distribution diagram of domains and mutations associated with lethal congenital contracture syndrome 10 (LCCS10, OMIMZ# 617022) and arthrogryposis, Perthes disease, and upward gaze palsy (APUG,OMIMZ# 6142622) in serine/threonine-protein kinase *NEK9*. RCC1, RCC1-repeats; CC, coiled-coil domain; and LIR, LC3-interacting region.

Sanger sequencing results of the two families and the diagram of domains and mutations in *NEK9* associated with skeletal dysplasia are present in Figure 2. A summary of phenotypic and genotype features of all reported patients involving the *NEK9* gene is shown in Tables 1, 2.

**TABLE 1:** Summary of phenotypic features of all reported patients.

Origin	Patient	GA weeks	Sex	Developmental index	SGA	Age	Clinical l manifestation	PMID
Consanguineous Irish Traveller family A	1	28^+4^ dead	M	BW: 892 (< 3rd) HC: 25 (9th) L: NA	+	—	Shortening of all four limbs, contractures of all major joints, micrognathia, short neck with reduced flexion, long overlapping fingers, adducted thumbs, and narrow hips with right-sided hydrocele	26908619
	2	21^+1^ TOP	M	BW: 260.85 g HC: 17.5 cm L: NA	+	—	Shortening of all four limbs, contractures of all major joints, long narrow head, long philtrum, high arched palate, micrognathia, neck short and stiff, overlapping fingers, adducted thumbs, hips and shoulders internally rotated, and appearance of pterygia secondary to severe contractures	
Consanguineous Irish Traveller family B	3	26^+2^ SB	F	BW: 630 g OFC: 23.3 (< 3rd) L:NA	+	—	Shortening of all four limbs, contractures of all major joints, small eyes, low-set ears, long philtrum, small chin, narrow palate, short neck with right torticollis, thoracic scoliosis, lateral deviation of fingers, and abnormal palmar creases	
	4	31 SB	F	BW: 890 g HC: 25.5 cm (< 3rd) L: NA	+	—	Shortening of all four limbs;, contractures of all major joints, beaked nose with a saddle bridge, long philtrum, macroglossia, sunken and downward-slanting eyes, and large omphalocele	
Consanguineous	5	Fetus		NA	NA	NA	Arthrogryposis multiplex congenita and fetal akinesia sequence	29096039
Consanguineous Saudi family	6 siblings	Term	F	BW: 3480 g (75th), HC: 35 cm (75–90th), L: 50 cm (50th)	-	12 years	Newborn examination showed limited hip abduction; later orthopedics confirmed multiple joint contractures involving the hands, elbows, hips, and knees; mild camptodactyly most pronounced in the left middle finger; mild atrial septal defect and mild pulmonary stenosis; uncontrolled bronchial asthma; severe atopic dermatitis in infancy; bilateral Perthes disease (avascular necrosis of the femoral head); upward gaze palsy; and normal cognitive development and normal growth parameters at 12 years old	21271645
	7 siblings	35	F	NA	—	15 years	Premature delivery at 35 weeks; newborn examination showed pulmonic stenosis and ventricular septal defect, pyloric stenosis (at 4 weeks of age); later in infancy, evaluation by pediatric orthopedics confirmed the presence of mild form of arthrogryposis involving the hands, elbows, hips, knees, and ankles; severe flexion deformity of the fingers; avascular necrosis of the right femoral head (at 2 years old); upward gaze palsy; uncontrolled bronchial asthma (since early childhood); obstructive sleep apnea (at 11 years old); IQ 85; and height 146 cm (< 3rd centile).	
Cousin of the two sisters	8	40	F	NA	—	7.5 years	Newborn examination showed pyloric stenosis and widespread limited mobility of joints; limited extension of elbows and knees, markedly reduced rotation and range of both hips; overriding toes; upward gaze palsy; subtle facial dysmorphism; two femoral heads and echocardiography was normal; normal cognitive development and normal growth parameters at 7.5 years old; and repeated episodes of bronchial asthma	
NA	9	Fetus	NA	NA		NA	Short long bones, bowed femur, clubfeet, deviation of hand, scoliosis, micrognathia, and abnormal filling stomach	32333414
Chinese Han	10	32^+2^	M	BW: 1680 g (< 3rd), HC: 30 cm (< 3rd), L: 40 cm (< 3rd)	+	2.5 months discharge	32^+2^ weeks, premature boy, IUGR; ventilator treatment after birth due to respiratory distress and pulmonary hemorrhage; newborn examination showed neck stiff, mild-moderate form of arthrogryposis involving the hands, elbows, and hips; his bilateral third, fourth, and fifth finger were camptodactyly; mild deformity of left toe; subtle facial dysmorphism; patent foramen ovale, mild pulmonary stenosis, mild-moderate pulmonary hypertension; and pyloric stenosis	This study
Chinese Han	11	34^+4^	M	BW: 2700 g (50–90th), HC: 33 cm (50–90th), L: 43 cm (10th)	_	2 months discharge	Newborn examination showed marked increase in muscle tone; atrial septal defect and mild pulmonary stenosis; moderate form of arthrogryposis involving the hands, elbows, hips, and knees; and tube feeding due to not sucking	This study

*GA, gestational age; SB, stillbirth; TOP, termination of pregnancy; BW, birth weight; L, length; HC, head circumference; SGA, small for GA; NA, not available.

**TABLE 2:** Summary of genotype features of all reported patients involved in the NEK9 gene.

Patient nos.	Gene	Inheritance	Diagnosis	Omimid	Mu type	HGVS DESCR	NM	Year	PMID
1	NEK9	AR	Lethal congenital contracture syndrome 10	#617022	Homozygous non-sense	c.1489C>T; p. Arg497* pathogenic	NM_033116.4	2016	26908619
2									
3									
4									
5	NEK9	AR	Lethal congenital contracture syndrome 10	#617022	Homozygous frameshift	c.1498delG; p.Glu500fs pathogenic	NM_033116.5	2018	29096039
6	NEK9	AR	Arthrogryposis, Perthes disease, and upward gaze palsy	#614262	Homozygous	c.2042G >A, p.Arg681 His	NM_033116.4	2015	26633546
7					Missense	Pathogenic			
9	NEK9	AR	Arthrogryposis, Perthes disease, and upward gaze palsy	#614262	Missense	c.1871A > G p. Asn624Ser pathogenic?	NM_033116.5	2020	32333414
					Frameshift	c.329_331delACA, p. Asn110del pathogenic?			
10	NEK9	AR	Arthrogryposis, Perthes disease, and upward gaze palsy	#614262	Non-sense	c.717 C > A, p.C239*741 likely pathogenic	NM_033116	2022	Novel, this study
					frameshift	c.2824delA, p.M942Cfs*21 likely pathogenic			
11	NEK9	AR	Arthrogryposis, Perthes disease, and upward gaze palsy	#614262	Non-sense	c.61 G > T p.E21* 959 pathogenic	NM_033116	2019	Novel, this study
					Frameshift	c.2824delA, p.M942Cfs*21 likely pathogenic			

### 4.2 Literature review

Keywords related to “*NEK9*” and “skeletal dysplasia,” “arthrogryposis,” “recessive,” “lethal congenital contracture syndrome 10,” and “arthrogryposis, Perthes disease, and upward gaze palsy” were used during the search in the Wanfang Data Knowledge Service Platform and Weipu Database (Chinese Journal Full-text Database covering time to August 2022), and no relative case was reported. The same keywords were used in PubMed (covering time to August 2022), and three publications were found containing eight cases of skeletal dysplasia; only six cases have been identified with pathogenic mutations in the *NEK9* gene, and genetic analysis was not carried out in another two cases. From HGMD (covering time to August 2022), we have found other two articles about skeletal dysplasia associated with *NEK9*.

In 2016, Casey et al. studied two Irish Traveller families with recessive lethal skeletal dysplasia. In the first family, there were two offspring with fetal akinesia, multiple contractures, shortening of upper and lower limbs, short broad ribs, narrow chest and thorax, pulmonary hypoplasia, and protruding abdomen. In the second family, three offspring had the same features, and one of them also exhibited bowed femurs. None of the affected babies survived: one baby died 1 h after birth, two babies were stillborn, one baby died *in utero*, and a fetus was induced by abortion. WES identified a novel homozygous stop-gain mutation in *NEK9* (c.1489C > T; p. Arg497*) in four affected fetuses (DNA was not available from the third fetus in the second family) (Casey Jillian et al., 2016).

In 2011, Alkuraya described a consanguineal Saudi family in which two sisters and one female cousin had almost the same disease, characterized by arthrogryposis, Perthes disease (avascular necrosis of the hip), and upward gaze palsy (APUG). They had multiple joint contractures including the elbows, hips, knees, and hands. Two had camptodactyly of the fingers, and one had overriding toes. At about 3 years, two children were diagnosed with avascular necrosis of the hip, and the third child had a mild collapse of the medial aspect of both femoral heads. Upward gaze palsy was apparent in childhood. All girls had bronchial asthma, and one had atopic dermatitis. Other features included pyloric stenosis in two girls, pulmonic stenosis in two girls, and a ventricular septal defect and small atrial septal defect in each one. Development was basically normal (Alkuraya et al., 2010). In 31 multiplex consanguineous families that appeared to have novel dysmorphology syndromes, including the Saudi family originally described by Alkuraya (2011), Shaheen et al. (2016) performed autozygome analysis followed by whole-exome and whole-genome sequencing. In the Saudi family with APUG, they identified a homozygous pathogenic missense mutation in *NEK9* (c.2042G > A, p. Arg681His) (Shaheen et al., 2015).

In 2018 and 2020, by parental exome sequencing in pregnancies, Stals and Deden (Stals et al., 2018; Deden et al., 2020) identified three mutations (c.1498delG and p.Glu500fs, associated with LCCS10 and c.1871A > G, p. Asn624Ser; c.329_331delACA, p. Asn110del associated with APUG) in *NEK9* in fetuses with congenital anomalies detected by ultrasound imaging. c.1498delG was identified as a pathogenic variant, and the other two were tentatively considered by the author to be of unknown significance.

## 5 Discussion

### 5.1 Humans have eleven different NEK (NIMA-related kinases) genes, named *NEK1* to *NEK11*

The NEK genes encode serine/threonine kinases, which play important roles in the regulation of the cell cycle and are involved in several cellular activities, such as centrosome separation, spindle assembly, chromatin condensation, nuclear envelope breakdown, spindle assembly checkpoint signaling, cytokinesis, cilia formation, and DNA damage response. The NEK9 gene (*location:* 14q24.3) contains 22 exons and encodes a member of the NIMA family of serine/threonine protein kinases NEK9 (NIMA-related protein kinase 9 with 979 amino acids). NEK9 is composed of an N-terminal protein kinase domain (residues 52–308), the central auto-inhibitory regulator of chromosome condensation 1 (RCC1)-repeats (residues 388–726), a C-terminal coiled-coil (CC) domain (residues 892–939), and a predicted LC3-interacting region (LIR) motif (residues 965–970) (Kalvari et al., 2014; Cullati et al., 2017; Yamamoto et al., 2021).

The conserved kinase domain is responsible for phosphorylating different substrates including histones, myelin basic protein, beta-casein, and BICD2. Phosphorylation of histone H3 on serine and threonine residues and beta-casein on serine residues is important for G1/S transition and S phase progression. *NEK6* and *NEK7* can be activated by *NEK9* phosphorylation (Belham et al., 2003; TanChin-Ming and Lee, 2003; Richards Mark et al., 2009), which is crucial in spindle formation and mitosis regulation. The C-terminal regions of the NEKs are highly divergent but have one relatively common feature that promotes autophosphorylation and activation (Fry et al., 2012). *NEK8* and *NEK9* can autophosphorylate in the non-catalytic C-terminal region to regulate their localization and/or activation (Bertran et al., 2011; Zalli et al., 2012).

Diseases associated with *NEK9* include nevus comedonicus (OMIMZ# 617025) (Levinsohn et al., 2016); lethal congenital contracture syndrome 10 (LCCS10, OMIMZ# 617022) (Casey Jillian et al., 2016); and arthrogryposis, Perthes disease, and upward gaze palsy (APUG, OMIMZ# 6142622 (Alkuraya et al., 2010). Reports related to *NEK9* gene variants are very limited. To date, five pathogenic mutations have been reported on the HGMD^®^ home page (cf.ac.uk) (Figure 2), including two cases of prenatal diagnosis, four cases of stillbirth, and two cases of children. Diagnosis names include LCCS10 and APUG.

LCCS10 is an autosomal recessive disorder characterized by degeneration of anterior horn neurons, extreme skeletal muscle atrophy, and congenital non-progressive joint contractures. The contractures can involve the upper or lower limbs and/or the vertebral column, resulting in varying degrees of flexion or extension limitations that are evident at birth (Casey Jillian et al., 2016). APUG is an autosomal recessive, syndromic form of arthrogryposis, a disease characterized by persistent joint flexure, upward gaze palsy, and avascular necrosis of the hip (Alkuraya et al., 2010; Shaheen et al., 2015).

In this study, patient 1 presented a mild-moderate form of arthrogryposis involving the hands, elbows, hips, and knees; stiff neck; camptodactyly of the fingers; and subtle facial dysmorphism, atrial septal defect, patent ductus arteriosus, and pyloric stenosis. Genetic analysis identified the compound heterozygous variation in NEK9: c.717 C > A (p.C239*741) and c.2824delA, p.M942Cfs*21. The c.717 C > A (p. C239*741) resulted in premature termination of protein translation and elimination of the final 741 amino acids. The mutation of c.2824delA located before the last domain of the peptide chain (LIR, Figure 2), is presumed to have some effects on the stability of protein structure. These two variants are consistent with disease co-segregation in families. Patient 2 presented with a mild-moderate form of arthrogryposis involving the elbows, hips, and knees, and he had an atrial septal defect and mild pulmonary stenosis. Genetic analysis revealed that he carried a compound heterozygous variation in *NEK9*, where one variation was the same as that of patient 1 (c.2824delA and p.M942Cfs*21) and the other was c.61 G > T, p. E21*959; this mutation causes premature translation termination, thereby affecting their function.

The two Chinese Han neonates’ clinical presentation was almost consistent with APUG; however, the patient is still young, and other manifestations such as Perthes disease and upward gaze palsy need further observation. It is worth noting that moderate persistent pulmonary hypertension was observed in patient 1. We speculated that the cause of pulmonary hypertension was pulmonary stenosis. This is a new feature that has not been reported before. Patient 1 can be bottle-fed on his own currently, and with the help of a physical therapist, his joint contracture has been relieved. It is a pity that patient 2 in Anhui Province was lost to follow-up after being discharged.

According to the aforementioned literature, variants in *NEK9*, which are associated with LCCS10, were homozygous nonsense and frameshift, and variants associated with APUG are either homozygous missense mutations or compound heterozygous mutants, such as the two cases in this study. In other words, different types of mutations in the same gene lead to different phenotypes. Patients with homozygous nonsense mutations or frameshift mutations will have severe phenotypes, while patients with homozygous missense mutations or compound heterozygous mutations may have mild symptoms.

What is the mechanism of the aforementioned diseases caused by *NEK9* gene mutation? Some researchers have suggested that it could be related to ciliary disorders. Casey (2016) not only found patients with lethal skeletal dysplasia fibroblasts that displayed a significant reduction in cilia number and length, but also provided evidence of the *NEK9* ortholog in C. elegans, nekl-1, which was almost exclusively expressed in a subset of ciliated cells, a strong indicator of cilia-related functions. Casey suggested that the lethal skeletal dysplasia caused by *NEK9* mutation may represent a novel ciliopathy (Casey Jillian et al., 2016). The LIR motif on *NEK9* C-terminal is conserved in land-living vertebrates and functions in primary cilia formation by mediating the *NEK9*–GABARAP interaction and *MYH9* degradation (Yamamoto et al., 2021). Dysfunctions in non-motile cilia are characterized as renal anomalies, skeletal anomalies, brain malformations, hepatic disease, hearing loss, anosmia, retinal dystrophy, central obesity, and facial anomalies (Reiter and Leroux, 2017). Critically, all the truncated mutant proteins in reported patients lack LIR motif, which may partly explain the complex and changeable clinical symptoms caused by *NEK9* mutations.

In summary, we identified the *HEK9* pathogenic variant in two Chinese neonatal infants with arthrogryposis. By comparative analysis of literature reports, we propose different types of mutations in *NEK9*, which will lead to different phenotypes. Patients with homozygous non-sense mutations or homozygous frameshift mutations in *NEK9* will have severe phenotypes (LCCS10), while patients with homozygous missense mutations or compound heterozygous mutations may have mild symptoms (APUG). With more and more case reports, the *NEK9* gene could be better understood. Our findings expand the genotype–phenotype knowledge of *NEK9*-associated arthrogryposis and provide evidence for further genetic counseling.

## Data Availability

The datasets presented in this study can be found in online repositories. The names of the repository/repositories and accession number(s) can be found in the article/Supplementary Material.
